# How efficient are specialized public health services in China? A data envelopment analysis and geographically weighted regression approach

**DOI:** 10.3389/fpubh.2025.1481402

**Published:** 2025-02-12

**Authors:** Qian Bai, Lieyu Huang, Yan Guo, Xin Xu, Zhouyixin Zhang, Yuan Wang, Hao Chen, Ying Bian

**Affiliations:** ^1^State Key Laboratory of Quality Research in Chinese Medicine, Institute of Chinese Medical Sciences, University of Macau, Macao, Macao SAR, China; ^2^Office of Labor and Social Security, School of Management, Tianjin University of Traditional Chinese Medicine, Tianjin, China; ^3^Office of General Administration, Chinese Center for Disease Control and Prevention, Beijing, China; ^4^Department of Public Health and Medicinal Administration, Faculty of Health Sciences, University of Macau, Macao, Macao SAR, China; ^5^Office of Policy and Planning Research, Chinese Center for Disease Control and Prevention, Beijing, China

**Keywords:** specialized public health facilities, efficiency, data envelopment analysis, geographically weighted regression, China

## Abstract

**Background:**

The Chinese public health system is grappling with escalating demands, which stemmed from the challenges of preventing chronic and infectious diseases, as well as the aging population. Meanwhile, in the context of restricted public health resources, how to efficiently utilize these resources becomes a paramount concern.

**Objective:**

This study aimed to evaluate the technical efficiency of specialized public health facilities, the major providers of public health services in China, then discuss its temporal and spatial distribution characteristics and finally investigate its influencing factors.

**Methods:**

The super slacks-based measure data envelopment model was constructed to calculate the efficiency of specialized public health facilities of 31 provinces from 2017 to 2019. Stepwise regression was applied to sort out significant independent variables. Then, geographically weighted regression was used to analyze the spatially varying associations between efficiency and independent variables.

**Results:**

On average, the average technical, pure technical and scale efficiencies were 0.6569, 0.7336 and 0.9206, respectively. Notably, a subtle downward trend was observed in the technical efficiency, which declined from 0.6889 in 2017 to 0.6238 in 2019. From the efficiency decomposition, this reduction was mainly caused by the decreasing of scale efficiency. Besides, substantial geographic variations were observed, with the eastern region exhibiting greater levels of technical and pure technical efficiency. Contrarily, the western region appeared to perform better in terms of scale efficiency. Based on the geographically weighted regression, the proportion of public health expenditure had a noticeable negative impact on the technical efficiency, especially in partial central and eastern coastal provinces. On the other side, the ratio of older population, the sex ratio and the Nitrogen Oxides emission volume had positive impacts on technical efficiency with variations in coefficient magnitude across different geographic areas.

**Conclusion:**

The efficiency of specialized public health facilities has not achieved the optimal status, particularly in terms of the pure technical efficiency. Moreover, the geographic variation was a significant issue affecting the sustainable and balanced performance of public health delivery system in China. The spatially heterogeneous associations between macro-regional factors and efficiency provide in-depth insights in assisting local governments to formulate more targeted and effective interventions, thereby contributing to reduce regional disparities.

## Introduction

Achieving universal health coverage (UHC) is one of the political goals of national governments, a state of all members accessible to necessary health services without any financial hardship. Although impressive progress has been made toward UHC till 2015, it has been stagnated in recent years (1). As estimated by WHO, up to 61% of the global population have no access to necessary health services in 2030, which is an obvious deviation from the UHC target (1, 2). Optimizing the performance of health system is recognized as a feasible, affordable and effective pathway for achieving UHC (3). In the health system, curative medical system and preventive public health system coordinate their efforts for improving the overall wellness, with public health gaining increasing attention in the wake of the recent global pandemic (4). Public health system provides a range of interventions, such as health education, immunization and improving environmental sanitation for the sake of physical, mental and social well-being of population in a broader sense (5). According to a recent study, there exist considerable potentials for improving the performance of public health sector, which could exert substantial positive impacts on the overall efficiency of health system (6). Therefore, it is of great importance and urgency to promote the performance of public health agency in order to maximize its contributions to UHC.

**Figure 1:**
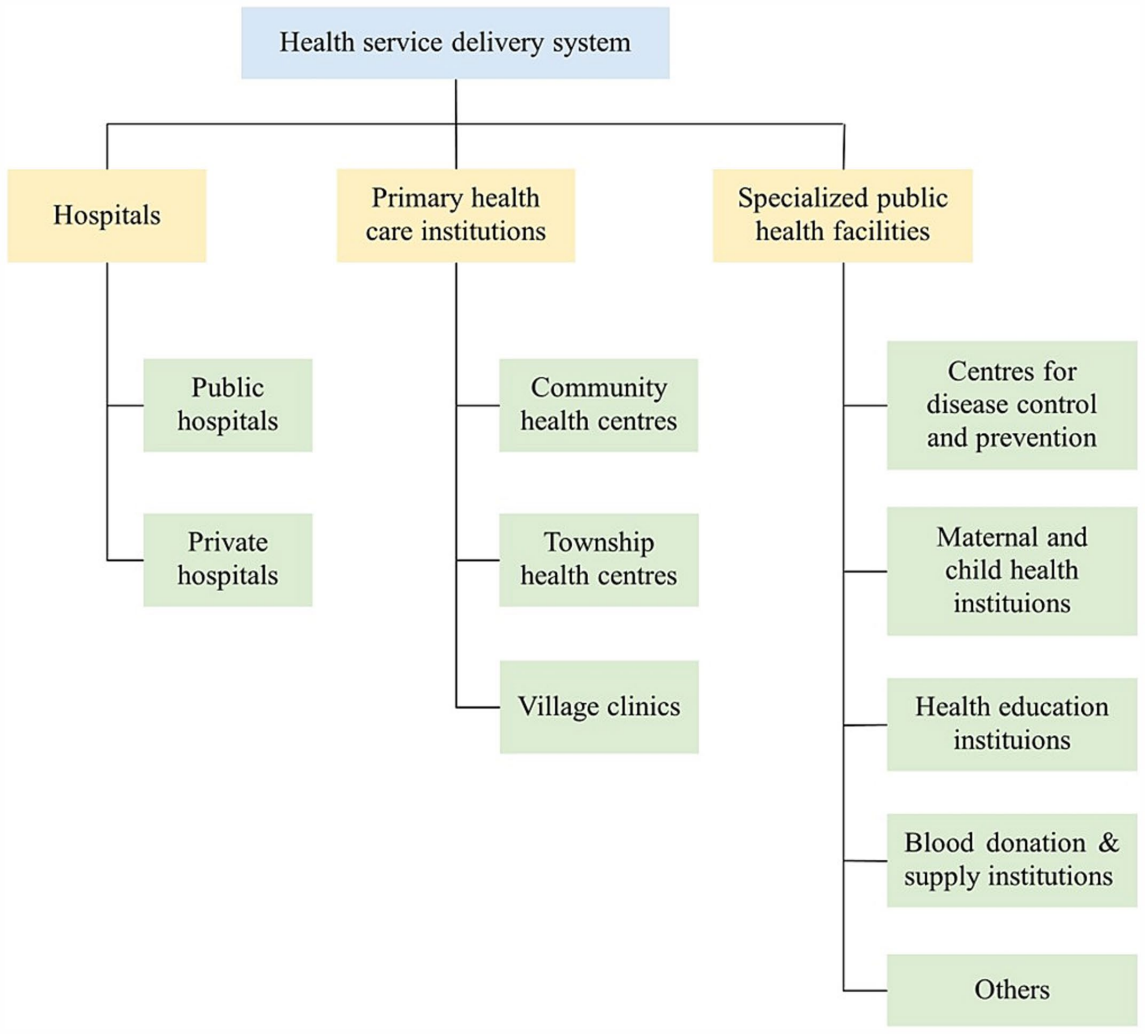
Framework of health service delivery system in China.

The structure of public health system differentiates by nations, including its responsible bodies and functions. In China, the healthcare delivery system comprises three sub-sectors, which are hospitals, primary health care institutions, and specialized public health facilities (Figure 1) (7). Hospitals deliver emergency, outpatient and inpatient services. Primary health care institutions are largely responsible for common and frequently-occurring diseases of local residents, as well as basic public health services. And specialized public health facilities are the major providers for public health services, including prevention of infectious and chronic diseases, management of maternal and child hygiene, health education and others. However, due to the disappearance of large-scale pandemic for years, together with the political inclination of “emphasizing medical treatment and neglecting prevention” in China, the role of specialized public health facilities has not been taken serious enough till the outbreak of SARS in 2003 (8, 9). Furthermore, its development has also been impeded by a number of practical issues, such as insufficient financial supports, personnel instability and fragmented public health system (10, 11). The roles of specialized public health entities have been further emphasized due to the prevalence of COVID-19. In this context, there are significant theoretical and practical values in measuring and promoting the performance of specialized public health entities in China.

Moreover, China is a heterogeneous country in many aspects, including health resource and delivery. Narrowing down the regional differences in health services has always been one priority of national policies, such as health reform in 2009 and Healthy China 2030. Meanwhile, health policies are generally formulated in a top-down manner in China. In other words, the central government develops and enforces regulations and policies related to health system, while the local government puts them into practice. However, few research has dug into the performance of specialized public health facilities and its geographical differences, which is valuable for policy-decision. In this study, we aimed to address the following questions: what is the technical efficiency of specialized public health facilities in China? Does the efficiency have any regional/provincial differences? Among social and environmental factors, which one has the great impact on the technical efficiency at provincial level?

## Literature review

### Research on efficiency assessment model in health care system

Revealed by the systematic review, data envelopment analysis (DEA) is the most common approach for assessing efficiency in the health care sector (12). It is a non-parametric technique for measuring the relative efficiency of various entities (referred to as decision making units, DMUs) based on multiple inputs and outputs. The DMUs are sets of homogenous samples, which could be the health system (13, 14), hospital (15, 16), or one specific department/service (17, 18). For instance, Gavurova et al. (14) assessed and compared the national health systems’ performance across OECD countries. While, Akkan et al. (18) took the emergency departments in hospital as the DMUs for efficiency assessment by means of DEA.

There are numerous options available for the specific DEA model. The conventional DEA models indicate to Charnes-Cooper-Rhodes (CCR) and Banker-Charnes-Cooper (BCC) models, which remain the predominating choice for health care efficiency (12). Several pitfalls in the conventional models have gradually been noticed, meanwhile, some derived DEA models emerge and are increasingly adopted in researches. Ferreira et al. (19) applied the non-radial directional distance function model to evaluate the performance of public hospitals in Portugal. This approach takes into account both radial and slack improvements, rather than solely focusing on radial improvement as seen in conventional models. In another study, slacks-based-measure (SBM) DEA model was employed to evaluate the efficiency of Spain hospitals (20). Apart from considering the radial and slack improvements, the SBM DEA is capable of dealing with undesirable outputs. However, in most cases, plural DMUs are estimated as technically efficiency, abovementioned models fail to further rank these efficient DMUs. To overcome this limitation, some scholars utilized super-efficiency DEA model to evaluate efficiency of medical health services (21).

Besides, more complexed DEA models have been developed by integrating diverse derivative models, thereby augmenting their advantages. Thereinto, the super SBM DEA model, which combines the merits of both the SBM and super DEA models, has recently become the preferred choice for efficiency evaluation in many fields, including health care (22), environment (23), urban development (24), and logistics industry (25). As for the Chinese health system, Sun et al. conducted a super SBM DEA model to explore and compare the efficiency of health care services across 31 provinces. Similarly, Zhao et al. employed the same approach to measure the performance of primary health care institutions in China. After analyzing the distinctive features and applications of various common DEA models, we determined to construct a super SBM DEA model in our study for measuring the efficiency of specialized public health facilities.

### Research on the efficiency of public health system

In practice, most researchers concentrate on the efficiency assessment for medical services (12, 26), while attention are comparatively inadequate for preventive public health services. From a global perspective, there are significant geographical disparities in the performance of public health systems, for instance, public health systems in the European region are more efficient than those in the African, South-East Asia, and Western Pacific regions (27). By means of DEA, Mukherjee K et al. found that public health sectors operated with nearly 28% inefficiency in the US (28), and emphasized the significance of internal funding for solving this inefficiency. Similarly, there was 24% inefficiency in the public health sectors of Senegal (29). Besides, a recent study evaluated the performance of the unintentional childhood injury intervention, one public health program in Japan, which presented with increasing trends and shrinking regional disparities (30).

Similarly, the performance of specialized public health facilities, the principal supplier for public health services in China, has been less extensively studied compared to that of hospitals and primary health care institutions (31–37). By employing the traditional DEA model, Dong LM and Zu JT discovered that specialized public facilities demonstrated a lower level of efficiency in contrast with primary health care facilities and hospitals in China from 2009 to 2017 (31). Using the same approach, scholars have reported the inefficiency of 56% and 55.6% in specialized public health facilities in 2015 and 2019 (38, 39), indicative of remarkable underutilization of available public health resources. Besides, some assessed the efficiency of certain specialized public health institution, such as centers for disease control and prevention (40) and maternal-child health institutions (41). So far, only a few studies measured the efficiency of specialized public health facilities in China, and mostly employed the traditional DEA model.

### Research on the influencing factors for estimated efficiency in health care system

Understanding the determinants of efficiency could provide empirical evidence for policy formulation. Given limited researches exploring the determinants affecting public health efficiency, this subsection provides a review of the methods and findings related to health care system.

It has been extensively discussed how demographic, social, and economic factors affect the efficiency of health systems. For example, urbanization rate was found to be positively associated with technical efficiency of primary health care system in China (42). A higher proportion of vulnerable population, like children or older adults, tended to reduce the efficiency of health care system (2). The relationships between different sources of health spending and health system efficiency have empirically been recognized (43). Besides, the environmental elements are associated with population wellness, which may affect the performance of public health system (44).

Econometric models, including Tobit regression (45, 46), bootstrap truncated regression (47), and multiple linear regression (48), are usually employed to investigate the potential determinants for health system efficiency. However, these models estimate the regression parameters as a whole, failing to distinguish the unique impacts on different sub-regions within a single regression analysis. In a prior Chinese study, it constructed four Tobit regression models to analyze the determinants for rural public health efficiency from perspectives of national, eastern, central and western regions, and remarkable differences were noted in the regression results. Specifically, the economic level was remarkably associated with the rural public health services in eastern and western regions, not the central region (49). This finding confirms our concern that a specific determinant may have distinct impacts in different geographic regions.

The geographically weighted regression (GWR) model enables the parameter estimation from the global and local spatial scales, identifying the significant determinants for each geographic unit. Several scholars constructed GWR model to identify the spatially varied associations between socio-economic factors and health resource allocation in China (50, 51). Therefore, we employed the GWR model in our research to in-depth understand the spatial heterogeneity of factors affecting public health facility efficiency, which also extends the application of GWR approach in health system efficiency.

## Materials and methods

### Input and output variables

For efficiency assessment in health system, labor-, capacity- and expense-related indicators are generally selected as inputs, and activity-related indicators constitute the outputs (26). Health technicians which involve doctors, nurses and pharmacists, make up nearly 80% of total personnel and handle the majority of services in specialized public health facilities (38, 39). For this reason, the number of health technicians per thousand people is chosen as the proxy for labor input. Besides, we identified the number of beds per thousand people in professional public health services as the capacity-related input (52, 53). Also, we noted that the expense-related variables had been used in efficiency evaluation for hospitals, primary health institutions and other health services (12, 26, 54, 55), therefore, we involved the operating cost per capita of specialized public health facilities as another input indicator in our study.

In the context of China, specialized public health facilities are primarily responsible for the prevention and management of infectious and chronic diseases, maternity and child hygiene, epidemic surveillance and health education (8). In correspondence to their primary mission, several outputs have been frequently chosen for measuring the efficiency of public health services, including infectious incidence rates of category A and B, maternal health management rate, under-three child health management rate and the number of health education (38, 39). However, the number of health education is more like an intermediate outcome for the former three indicators, which we thought is unreasonable to be listed as one output. Therefore, we confirmed three output variables for specialized public health facilities, namely infectious incidence rate of category A and B, maternal health management rate and under-three child health management rate, which comply with the officially regulated major goals of such agency (7, 56).

**Figure 2:**
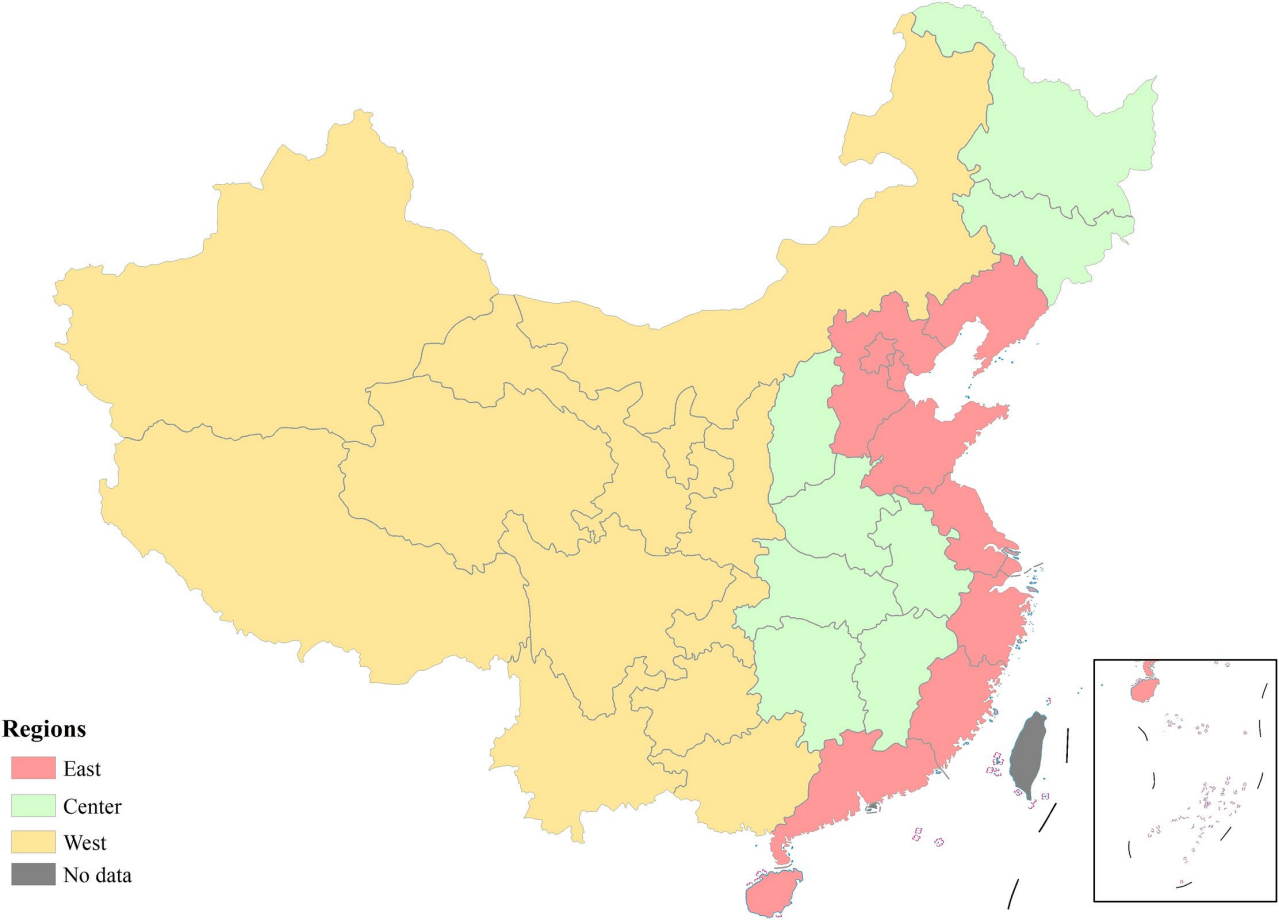
Study sites in this research. The region division is accordance with China Health Statistics Yearbook. This figure was drawn based on the standard map [No. GS (2019)1822] from Ministry of Natural Resources of the People’s Republic of China.

Altogether, we included three input and three output variables. And 31 provinces were treated as the DMUs in our study (Figure 2). Input and output variables were retrieved from National Health Financial Annual Report (each specialized public health facilities is required to annually submit key statistics to an electronic system, and we retrieved operating cost per capita of specialized public health facilities from this system) and China Health Statistics Yearbook (57). The detailed statistics of indicators are depicted in Table 1.

**Table 1:** Summary statistics of input, output, and independent variables of 31 provinces in China, 2017–2019.

Variables	Mean	SD	Min	Max
Input^a^				
Number of health technicians per thousand people	48.12	10.91	23.73	74.91
Number of beds per thousand people	17.60	7.32	4.00	32.16
Operating cost per capita (Yuan)	132.77	43.82	60.29	299.68
Output				
Infectious incidence rate of category A and B^b^ (%)	236.12	100.90	111.54	659.75
Maternal health management rate (%)	89.24	8.23	54.4	99.9
Under-three child health management rate (%)	91.49	4.67	71.30	98.00
Independent variables (*n* = 17)				
GDP per capita (Yuan)	65114.76	29748.15	28497.00	164220.00
Urbanization rate (%)	59.94	11.69	30.89	88.30
Population per km^2^	480.45	756.22	2.81	4186.21
Sex ratio^c^ (%)	104.50	4.57	96.73	123.18
Ratio of children population/0–14 yrs. (%)	16.73	4.09	9.84	26.07
Ratio of older population/>65 yrs. (%)	11.37	2.50	5.68	16.26
Disposable income per capita (Yuan)	28244.25	11529.07	15457.30	69441.60
Engle’s coefficients (%)	28.92	4.05	19.72	46.40
Percentage of illiterate population aged ≥15 yrs. (%)	5.77	5.84	1.23	35.23
Proportion of public education expenditure^d^ (%)	15.66	2.54	10.99	20.41
Total volume of Sulfur Dioxide emission (Ton)	170348.41	115715.88	1923.00	433093.00
Total volume of Nitrogen Oxides (NO_X_) emission (Ton)	416203.05	286556.02	34095.00	1227933.00
Total volume of Particulate Matter emission (Ton)	376951.45	236477.44	15386.00	1060885.00
Proportion of public health expenditure^e^ (%)	8.01	1.37	5.43	10.44
Current health expenditure (proportion of GDP, %)	7.40	1.88	4.01	11.97
Current health expenditure per capita (Yuan)	4481.65	1883.18	1010.09	13766.77
Out-of-pocket health expenditure (proportion of current health expenditure, %)	27.27	5.78	5.16	35.18

a The input is statistics data for specialized public health facilities.

b Category A includes plague and cholera, and category B has 26 infectious diseases.

c Sex ratio: male population/female population.

d Proportion of public education expenditure out of general public expenditure.

e Proportion of public health expenditure out of the general public expenditure.

### Independent variables

Since few studies investigate the influential factors for the performance of public health services, we identified several independent variables associated with health system efficiency (2, 42–44). The independent variables were collected from China Statistical Yearbook (58) and China Statistical Yearbook on Environment (59). Summary statistic for all independent variables is presented in Table 1.

### The super slacks-based measure model with undesirable output

The DEA model has been widely used in various fields since its initial proposal in 1978 (2). The underlying principle of DEA is to construct an optimal production frontier through a linear programming approach. Unlike parametric methods, DEA does not require the pre-specification of any functional form. Besides, it can tackle with multiple inputs and outputs, which also makes DEA stand out among a variety of efficiency evaluation methods. As mentioned before, the conventional CCR and BCC models only assess the radial efficiency of DMUs, which means that the inefficient DMUs is assumed to improve their performance through proportional reduction in the inputs or proportional increase in the outputs. It disregards the slack improvements existing in input or output variables, which possibly leads to an overestimation of the actual efficiency. Moreover, it is quite difficult to control the radial improvements in input and output variables in most practices. Therefore, the non-radial DEA models has been increasingly applied in researches of efficiency evaluation, which could directly handle the potential slack improvement and avoid the radial deviations. Apart from this, the SBM DEA model, belonging to the non-radial model, has been empirically proved to exhibit excellent performance in dealing with undesirable outputs and applied into a mass of academic researches (22–25, 60).

According to the principle of conventional DEA model, when one DMU states in the optimal production frontier, indicating achieving fully efficient with the maximum value of 1. In most cases, plural DMUs have the “efficient status.” Thus, it is meaningful to further distinguish the efficiency among these efficient DMUs. In 2002, Tone proposed an SBM of super-efficiency (hereafter referred to super SBM DEA model), which is capable of ranking the efficient DMUs (61). The infectious incidence rates of category A and B is an undesirable output variable, therefore the super SBM DEA model is selected in our research to handle the issues of undesirable output, slack improvements and ranking efficient DMUs.

Let us assume n DMUs in our research. Each DMU produces $s_{1}$ desirable outputs and $s_{2}$ undesirable outputs by means of m input variables. According to Tone (60), the SBM model with can be specified as follows in this case:


$$\rho_{k}=\min\frac{1-\frac{1}{m}\sum_{i=1}^{m}s_{i}^{-}/x_{ik}}{1+\frac{1}{s_{1}+s_{2}}\left(\sum_{r=1}^{s_{1}}S_{r}^{g}/y_{rk}^{g}+\sum_{t=1}^{s_{2}}S_{t}^{b}/y_{tk}^{b}\right)}$$

Subject to


$$x_{k}=X\lambda +S^{-}$$

$$y_{k}^{g}=Y^{g}\lambda -S^{g}$$

$$y_{k}^{b}=Y^{b}\lambda +S^{b}$$

$$S^{-}\geq 0,S^{g}\geq 0,S^{b}\geq 0,l\leq e\lambda \leq u,\lambda \geq 0$$

Where λ denotes the weight vector; $S^{-}$, $S^{g}$, and $S^{b}$ indicated the slacks of inputs, desirable outputs and undesirable outputs, respectively. And $\rho_{k}$ is the technical efficiency of public health service of province k ranging from 0 to 1. If $\rho_{k}$ equals to 1, the $k_{th}$ DMU achieves the efficient status. Then, the super-SBM DEA model is applied to these efficient DMUs to further rank their efficiency values. The formula is specified as follows (61):


$$\rho_{k}^{*}=\min\frac{\frac{1}{m}\sum_{i=1}^{m}{\bar{x}}_{i}/x_{ik}}{\frac{1}{s_{1}+s_{2}}\left(\sum_{r=1}^{s_{1}}{\bar{y}}_{r}^{g}/y_{rk}^{g}+\sum_{t=1}^{s_{2}}{\bar{y}}_{t}^{b}/y_{tk}^{b}\right)}$$

Subject to


$$\bar{x}\geq \sum_{j=1,\neq 0}^{n}\lambda_{j}x_{j}$$

$${\bar{y}}^{g}\leq \sum_{j=1,\neq 0}^{n}\lambda_{j}y_{j}^{g}$$

$${\bar{y}}^{b}\geq \sum_{j=1,\neq 0}^{n}\lambda_{j}y_{j}^{b}$$

$$\bar{x}\geq x_{k},{\bar{y}}^{g}\leq y_{k}^{g},{\bar{y}}^{b}\geq y_{k}^{b},{\bar{y}}^{g}\geq 0,\lambda \geq 0$$

Where $\rho_{k}^{*}$ indicates the super efficiency of the $k_{th}$ DMU, whose value equals or exceed 1. And the others have the same meaning with abovementioned SBM DEA model with undesirable outputs.

Technical efficiency in DEA analysis refers to “the capacity or even the ability to make the most appropriate use of the resources available to achieve an intended result” (62). Specifically, the technical efficiency is measured under the assumption of constant return to scale. However, not every sample would be stated in the aforementioned status, and the technical efficiency would be influenced by sample’s scale. Hence, the technical efficiency is decomposed into pure technical and scale efficiency. Pure technical efficiency is correlated with managerial factors or technology level (e.g., advanced technology and equipment in health institutions), while scale efficiency implies whether the operation size of sample is optimal, too large or too small, which corresponds to constant-, decreasing- or increasing returns to scale, respectively (63). The relations between three types of efficiency can be specified as follows:


Technicalefficiency=puretechnicalefficiency∗scaleefficiency

And all the efficiency analysis was conducted in Max DEA software.

### Spatial autocorrelation analysis

Global Moran’s I index was applied to explore the spatial patterns of estimated technical efficiency of specialized public health facilities. The formula of Global Moran’s I was specified as follows (64):


$$I=\frac{n\sum_{i=1}^{n}\sum_{j=1}^{n}W_{ij}\left(X_{i}-\bar{X}\right)\left(X_{j}-\bar{X}\right)}{\sum_{i=1}^{n}\sum_{j=1}^{n}W_{ij}\sum_{i=1}^{n}{\left(X_{i}-\bar{X}\right)}^{2}}$$

Where, n denotes research samples (31 provinces), $W_{ij}$ represents the matrix of spatial weights and inverse distance weight matrix is adopted in this study to comply with Tobler’s First Law of Geography, $X_{i}$ and $X_{j}$ indicates the technical efficiency of province i and province j, respectively.

The value of I ranges from −1 to 1. If the value is equal to 0, no spatial autocorrelation exists; otherwise, positive (I > 0) or negative (I < 0) spatial autocorrelation exists. This procedure was performed in ArcGIS version 10.7.

### Stepwise regression for variable selection

To choose significant influential factors, multi-variate linear stepwise regression was constructed based on the Z-scores of the dependent variable (technical efficiency obtained from super-SBM DEA model), and all 17 independent variables in Table 1. These analyses were carried out using SPSS version 25.

### Geographically weighted regression

Although the traditional econometric regression model is usually used to capture the associations between dependent and independent variables. It takes no account of the spatial features of samples. In most cases, each observation contains some geospatial information; for instance, the 31 provinces in our research inherently carry geographical information, which is reflected in the varying distance between the provinces. The ignorance of spatial effects might lead to bias and inconsistency for the parameter estimation. Under this condition, several spatial econometric models, including spatial error model, spatial lag model and spatial Durbin model, have attracted intensive attention with the advantages of handling the spatial interdependency in error terms, dependent or independent variables. However, these spatial econometric models also belong to a global analysis, in another word, a unified parameter is developed for each explanatory variable (65). And the problem of spatial heterogeneity, referring to uneven distribution of measurements over space, still remained unsolved.

The GWR, proposed by Fotheringham, is a statistical technique to discover the spatially non-stationary relationships between dependent and independent variables (66). It embeds geographic characteristics into regression parameters, allowing for the estimation of a unique set of regression coefficients for every geographical unit. This distinctive capability enables GWR to capture local variations in influential factors, thereby assisting local governments in implementing more targeted and effective interventions, as opposed to one-size-fits-all approaches. Consequently, the GWR model was employed in our research to comprehensively identify the spatial heterogeneity between the explanatory variables and the estimated efficiency.

In practice, when spatial autocorrelation is detected, which means the occurrence of spatial non-stationarity, the GWR should be considered (67). The basic structure of GWR is (64):


$$y_{i}=\beta_{0}\left(\mu_{i},v_{i}\right)+\sum_{k}\beta_{k}\left(\mu_{i},v_{i}\right)x_{ik}+\varepsilon_{i}$$

where, $y_{i}$ is the technical efficiency of province i, $x_{ik}$ represents the k^th^ independent variable for province i, $\left(\mu_{i},v_{i}\right)$ describes the spatial coordinates of province i, $\beta_{0}\left(\mu_{i},v_{i}\right)$ and $\beta_{k}\left(\mu_{i},v_{i}\right)$ denote the regression coefficients, and $\varepsilon_{i}$ is the error term.

The spatially varied parameters are estimated relying on the spatial weight matrix between data points. In this study, corrected Akaike information criterion (AICc) is used to determine the optimal bandwidth and Gaussian kernel function is constructed to calculate the spatial weight matrix. The GWR analysis and mapping of coefficients were performed in ArcGIS version 10.7.

## Results

### Estimated efficiency of specialized public health facilities

As estimated, the average technical efficiency, pure technical efficiency and scale efficiency scores of specialized public health facilities were 0.6569, 0.7336 and 0.9206, respectively. Regarding the temporal trend, technical efficiency kept decreasing from 0.6889 in 2017 to 0.6238 in 2019, which was primarily attributed to a substantial reduction in the scale efficiency (Table 2).

**Table 2:** Efficiency of public health services provision by year and region.

Year	Region	Technical efficiency	Pure technical efficiency	Scale efficiency
2017	Average	0.6889	0.7093	0.9727
	Eastern	0.8480	0.8659	0.9846
	Central	0.6553	0.6826	0.9640
	Western	0.5654	0.5835	0.9676
2018	Average	0.6579	0.7328	0.9185
	Eastern	0.8269	0.9825	0.8698
	Central	0.6160	0.6435	0.9516
	Western	0.5308	0.5635	0.9411
2019	Average	0.6238	0.7587	0.8705
	Eastern	0.8186	1.0224	0.8608
	Central	0.5845	0.7023	0.8628
	Western	0.4715	0.5547	0.8844
Average		0.6569	0.7336	0.9206

There exist obvious geographic variations in the efficiency of specialized public health services. The technical efficiency and pure technical efficiency were the highest in the eastern region, followed by the central and western regions in all research years. Remarkably, the pure technical efficiency of the eastern region was nearly 1.5 to 2 times higher than that of the central and western regions. Since the elite public health resources, such as highly skilled professionals, advanced information systems, and cutting-edge management and technologies, are predominantly clustered in the eastern regions. Contrarily, the western region possessed a relatively higher scale efficiency, and experienced less reduction in the scale efficiency over years (Table 2).

**Figure 3:**
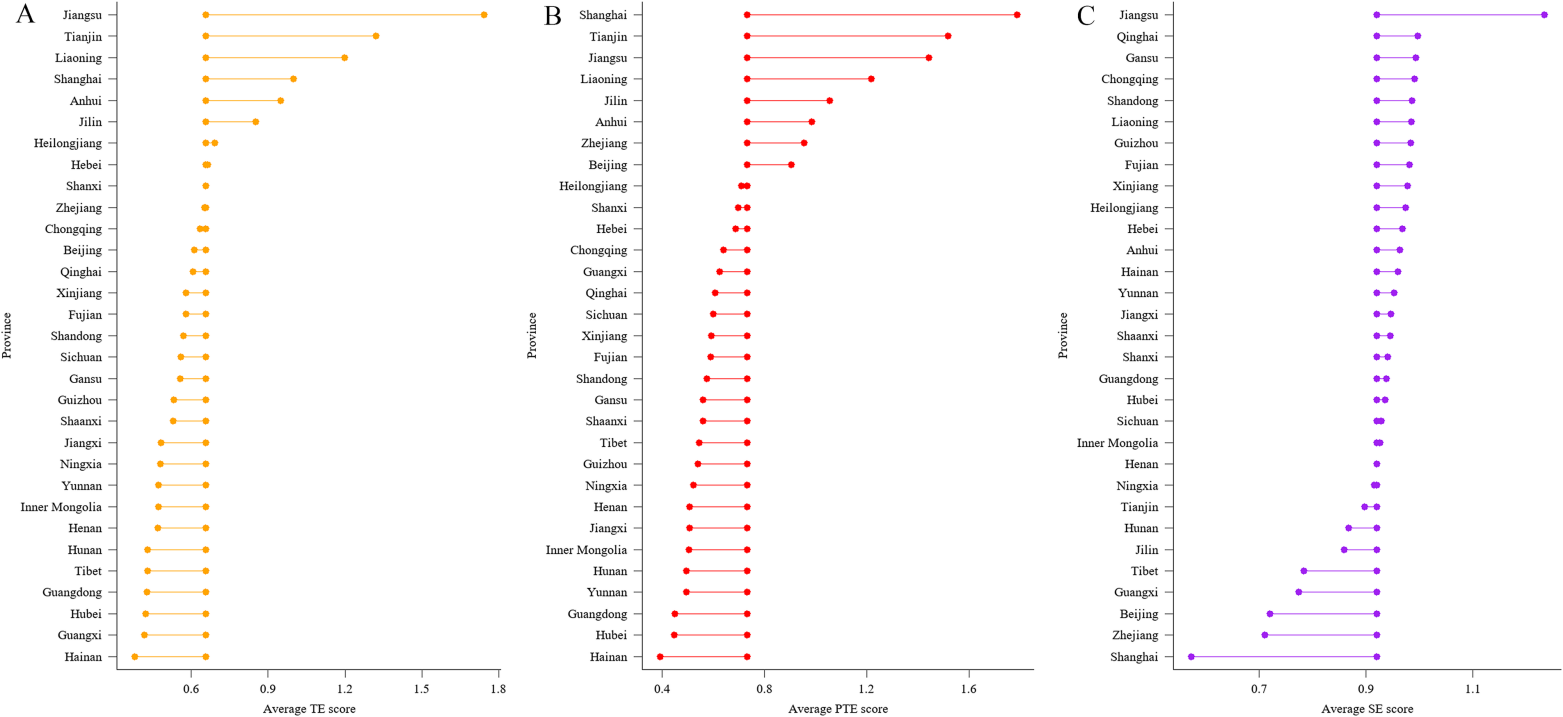
Average efficiency of public health services by provinces from 2017 to 2019. **(A)** technical efficiency; **(B)** pure technical efficiency; **(C)** scale efficiency. Centrally aligned data-points show the average TE score (0.6569), average PTE score (0.7336) and average SE score (0.9206) across 31 provinces from 2017 to 2019, with data-points to either side showing the average score for each province. TE: technical efficiency; PTE: pure technical efficiency; SE: scale efficiency.

The findings at the provincial level provided more insights into geographic variations (Figure 3; Supplementary Table S1). Merely one-quarter of the provinces surpassed the average score in terms of technical and pure technical efficiency, with the majority of them situated along the eastern coast, such as Jiangsu, Shanghai and Tianjin. This precisely unveiled the pressing issues in management and technology within Chinese public health system. Conversely, as regards the scale efficiency, a small fraction of provinces fell below the average score, particularly some economically developed provinces like Shanghai, Zhejiang and Beijing. This indicates that, for prosperous provinces, the emphasis of local governments should shift from continually expanding investment to adjusting the structure of existing resources, such as optimizing personnel quality and expenditure structure.

### Associations between independent variables and technical efficiency

Based on the stepwise regression, four variables were selected with a 95% confidence interval, which were ratio of older population, sex ratio, proportion of public health expenditure and total volume of Nitrogen Oxides (NO_x_) emission. Generally, a variance inflation factor (VIF) larger than 10 implies multi-collinearity. Since the largest VIF was merely 1.283, no multi-collinearity existed in the selected independent variables. The details for stepwise regression are depicted in Supplementary Table S2.

The GWR model is appropriate only when spatial correlation exists in the dependent variables. The global Moran’s I index revealed significant spatial correlations in the technical efficiency (Supplementary Table S3), suggesting the suitability and necessity of GWR.

As a result, the GWR regression was constructed between the technical efficiency and four independent variables. The details of the GWR regression are described in Supplementary Table S4 and Supplementary Figure S1. After the GWR regression, Global Moran’s I of the standard residual became insignificant, reaffirming the reliability of GWR regression (Supplementary Table S5).

According to the GWR regression, the proportion of public health expenditure and ratio of older population had a relatively stronger impact on public health services efficiency as a whole (Supplementary Table S4). During the research period, the proportion of public health expenditure negatively impacted public health efficiency. Its impact slightly decreased over time, whose absolute median value ranged from 0.4524 to 0.5480. A positive association was observed between ratio of older population and public health efficiency, with median coefficients changing from 0.4257 to 0.4793. Similarly, sex ratio was found to positively impact public health efficiency, whose impact reached the largest with a median coefficient of 0.3441 in 2018. The total volume of NO_x_ emission was the least influential factor with the median coefficient value from 0.1691 to 0.2773.

Subsequently, we displayed the coefficients into maps to explicitly exhibit the impacts of independent variables at provincial level.

### Ratio of older population

**Figure 4:**
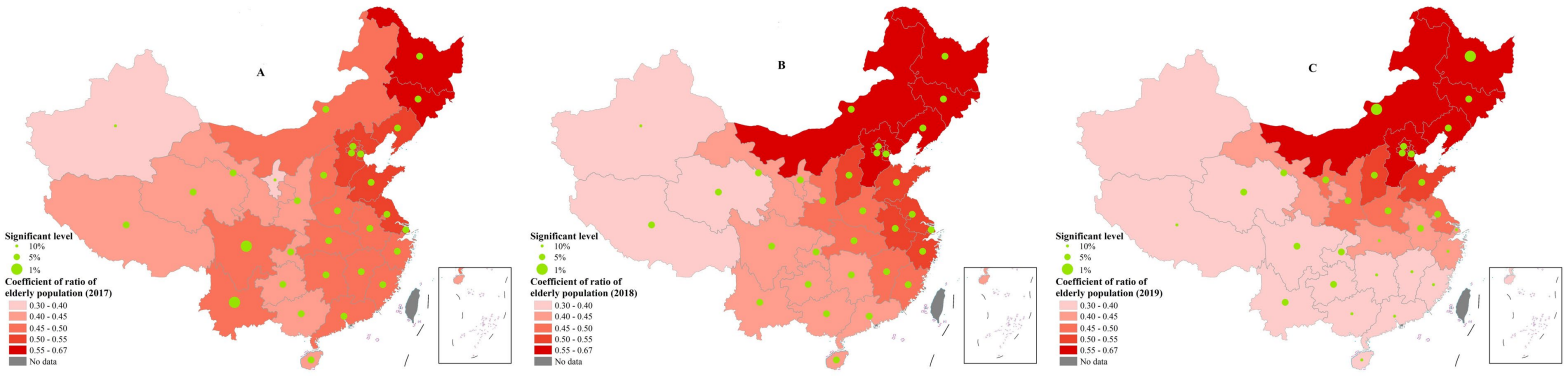
The spatial distribution of coefficients of ratio of older population. **(A)** 2017; **(B)** 2018; **(C)** 2019. These figures were drawn based on the standard map [No. GS (2019)1822] from Ministry of Natural Resources of the People’s Republic of China.

For the ratio of older population, its coefficient showed a descending trend from northeast to southwest regions. Specifically, ratio of older population had the highest impact on efficiency in Heilongjiang, Jilin and Liaoning all the time, followed by some other eastern provinces such as Beijing, Tianjin, Hebei and Inner Mongolia. Whereas the weakest association was found in provinces located in the west and the south regions including Xinjiang, Tibet and Hainan (Figure 4).

### Sex ratio

**Figure 5:**
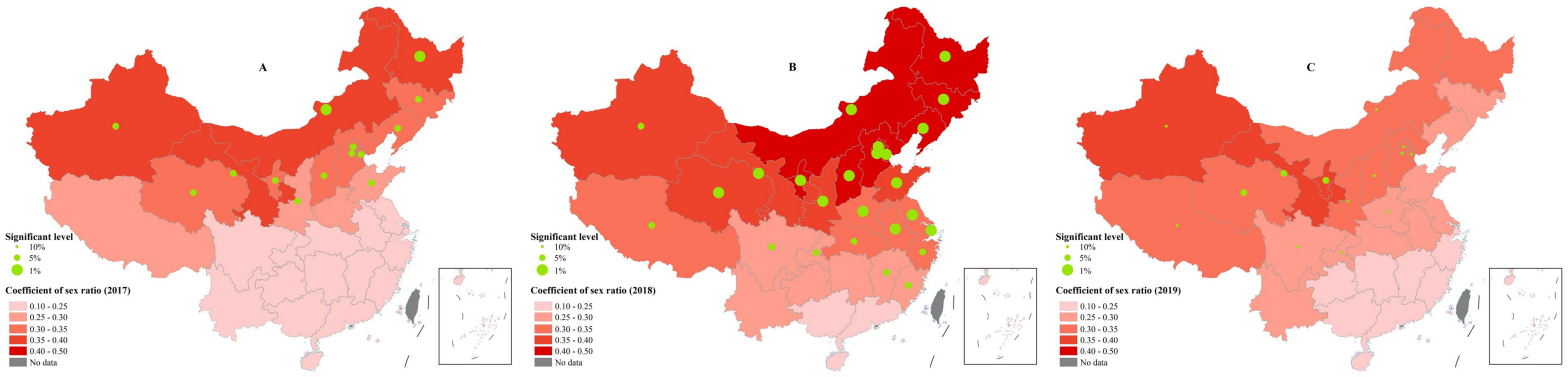
The spatial distribution of coefficients of sex ratio. **(A)** 2017; **(B)** 2018; **(C)** 2019. These figures were drawn based on the standard map [No. GS (2019)1822] from Ministry of Natural Resources of the People’s Republic of China.

The positive associations between sex ratio and efficiency gradually weakened from the north to the south. The coefficient of sex ratio was relatively higher in Inner Mongolia, Xinjiang, Gansu and Heilongjiang with a value of around 0.4; on the other side, the lowest coefficient appeared in some southern provinces especially Hainan, Guangdong and Guangxi with an approximate value of 0.2 (Figure 5).

### Proportion of public health expenditure

**Figure 6:**
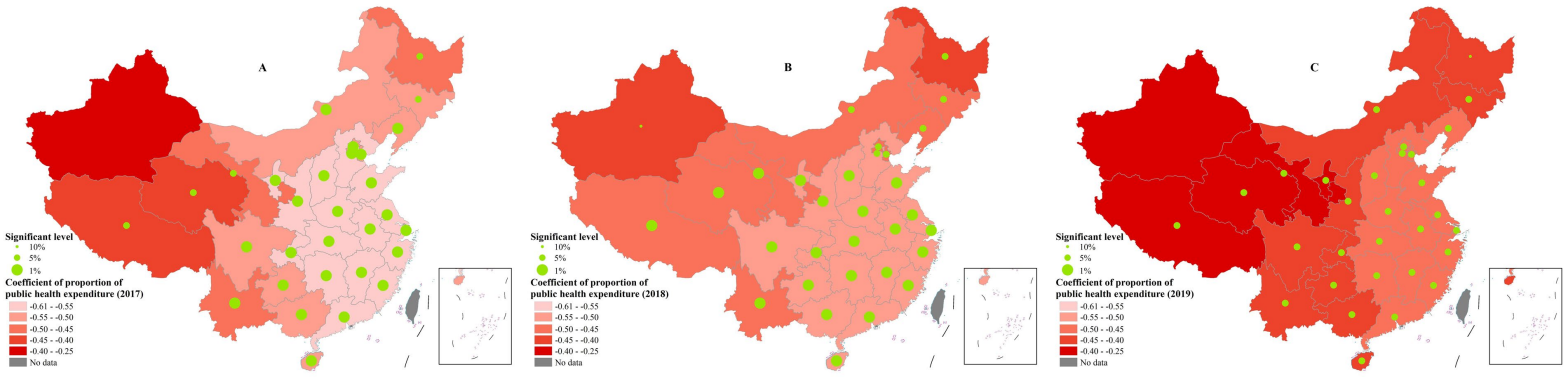
The spatial distribution of coefficients of proportion of public health expenditure. **(A)** 2017; **(B)** 2018; **(C)** 2019. These figures were drawn based on the standard map [No. GS (2019)1822] from Ministry of Natural Resources of the People’s Republic of China.

Different from the other three variables, a stronger negative association was identified between the proportion of public health expenditure and public health efficiency. No obvious geographical regularity was found in its coefficient distribution. In 2017 and 2018, the highest absolute coefficient primarily concentrated in central regions such as Shaanxi, Hubei, Henan, and Hunan, while it changed to eastern coastal provinces such as Fujian, Zhejiang, Shanghai, and Jiangxi in 2019. Besides, a comparatively weaker associations were found in provinces including Xinjiang, Tibet, Qinghai and Gansu, whose absolute coefficients ranged around 0.25–0.4 all years (Figure 6).

### Total volume of NOx emission

**Figure 7:**
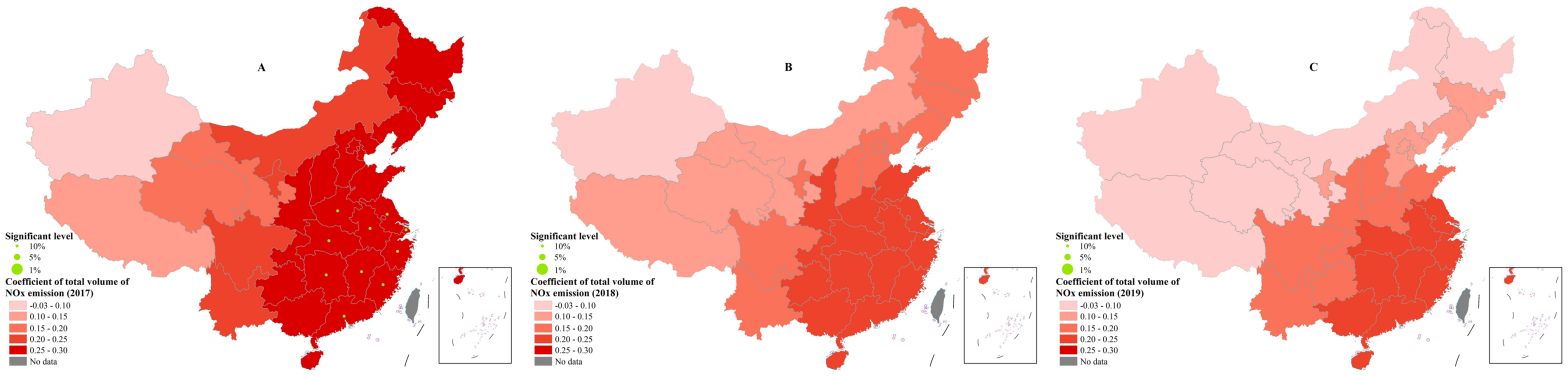
The spatial distribution of coefficients of total volume of NO_x_ emission. **(A)** 2017; **(B)** 2018; **(C)** 2019. These figures were drawn based on the standard map [No. GS (2019)1822] from Ministry of Natural Resources of the People’s Republic of China.

The association between total volume of NO_x_ emission and technical efficiency was the least among four independents. Notably, its impact gradually dampened from southeast to northwest. Higher coefficient was generally observed in provinces such as Guangdong, Hainan, Fujian and Jiangxi with a value of 0.2–0.3, whereas provinces with lower coefficients mostly distributed in the northwest region including Xinjiang, Gansu, Tibet and Qinghai (Figure 7).

## Discussion

This paper assessed the efficiency of specialized public health facilities, as well as its geographic characteristics and influential factors based on provincial data from 2017 to 2019. According to our findings, there existed substantial potential improvements for the technical efficiency of Chinese specialized public health facilities, especially in the pure technical efficiency. Meanwhile, prominent regional and provincial disparities were observed, which should be carefully considered by policy-makers. Furthermore, the geographically varied associations between social environmental factors and the technical efficiency may provide important guidance for central and local government in policy formulation.

The specialized public health services in China have not reached a state of full efficiency. The technical efficiency was 0.6569 on average, meaning that nearly 35% of available health resources was underused at that time. Similarly, scholars have already pointed out that a 28% inefficiency existed in American public health system (28), and nearly 80% of public health centers in Ghana operated inefficiently (68). From the technical efficiency decomposition, there was larger improving room for pure technical efficiency (26.64%) than scale efficiency (7.94%) in our study. Prior studies in China and Ghana have also drawn similar implications that, pure technical efficiency was the major obstacle for the performance of public health system (40, 68). As noted, pure technical efficiency is mainly related with the expertise and management level. From one side, staff aging and a shortage of highly educated professionals have become the predominant concerns for current public health system in China (69, 70). Meanwhile, professional public health facilities experience troubling brain drain, especially among younger employees, which is possibly caused by the asymmetry between lower salaries and heavy workloads (71, 72). All these together lead to the unsatisfactory consequence in the pure technical efficiency of specialized public health facilities. In 2020, the Chinese government issued a policy regarding cultivating highly-qualified public health talents in response to emerging public health events effectively and efficiently. It deserves extensive investigation and follow-up study whether this strategy could optimize workforce structure, and then facilitate the pure technical efficiency of public health system in China.

Surprisingly, the performance of specialized public health services showed a declining trend during the research period. In accordance with our findings, Pereira MA et al. also highlighted the global decline in public health index (27). Even though, the pure technical efficiency showed a rising trend in our study, which possibly benefits from technological progress, as well as improvements in inner management guided by official policies. In 2016, the State Council issued an outline for the “Healthy China 2030” initiative, highlighting the continuous improvements in public health system. For instance, it encourages the monitoring mechanism for infectious disease, which tends to optimize the performance for infectious disease management and control. In the following year, the National Health and Family Planning Talent Development Plan for the 13th Five-Year Plan Period emphasized bolstering the public health workforce in terms of quantity, quality, and structural optimization to address the increasing public health demands. All these policies contribute to the elevation in the management level and technology in public health system. Based upon our results, the decrease of specialized public health services’ efficiency was mainly driven by the backward movement of scale efficiency. All samples operated in a state of increasing- or decreasing- returns to scale, and the major issue transformed from undersize scale in 2017 to oversize scale in 2019 (Supplementary Table S6), which is square with one prior study (39). As government gradually highlighting the function of prevention, large quantities of resources, such as finance and labor, have been plunged into public health system (73, 74). Therefore, figuring out the ideal scale should take precedence over continuous investments for specialized public health facilities at present.

The specialized public health services efficiency showed evident regional and provincial disparities in our study. The eastern region obtained higher technical and pure technical efficiency followed by the central and the western region, which was consistent with previous studies (53). The vast geographic area of some western provinces might inhibit the efficient delivery of population-based public health services (75). On the other hand, since most public health services are provided without or at a small charge, local government takes the responsibility of providing financial supports for specialized public health facilities apart from central government subsidy (76, 77). As a result, the economically developed eastern region has more advantages over purchasing advanced equipment and absorbing skilled technicians by decent salaries, which leads to a higher pure technical efficiency in these areas. However, economic prosperity sometimes is detrimental to scale efficiency, which is mainly attributable to excessive investment in labor, equipment and other resources (78). This phenomenon has also been observed in this study, for example, the scale efficiency of some eastern developed areas, like Shanghai, Zhejiang and Beijing, ranked below the average score. Considering the striking regional disparities in specialized public health facilities’ efficiency, differentiated policies and actions should be made for specific regions. For less developed western and central regions where pure technical efficiency matters, the Chinese government could offer more financial support for introducing innovative technology and equipment, and attracting highly skilled health personnel (79); meanwhile, local facilities may seek cooperation with those well-developed agencies in the eastern region, such as staff training and assistance on optimizing inner administration. On the other side, the eastern region should pay attention to the excessive extension of investment, in case of wasting existing resources.

More importantly, spatial heterogeneity is considered when measuring the determinants on the technical efficiency of specialized public health facilities through GWR approach. As estimated, the proportion of public health expenditure had a large impact on public health efficiency in a negative manner. Liu T et al. also discovered financial government played a negative role for rural public health efficiency (49). Similarly, an earlier public health assessment in America revealed an inverse relation between government funding and efficiency (28). This could be interpreted in a couple of ways. From one perspective, public health organizations may function less effectively without too much financial pressure if they have been adequately supported by government subsidies. Besides, institutions with enough financial resources tend to expand their scale in terms of personnel, equipment, and buildings, which jeopardizes the scale efficiency as a result. Also, larger government support may reflect serious local public health problems, which helps to explain the strong negative associations with coastal provinces like Fujian, Zhejiang and Guangdong, which face a greater risk of national and international importation of infectious diseases (80).

Population structure was a potential driver for specialized public health efficiency, evidenced by the positive associations between older population proportion, female ratio and estimated technical efficiency. Vulnerable population such as the older adults, pregnant and children tend to be tortured by various ailments, and in turn generates more public health demands (81). Promoting the wellness of the older adults and female groups are the preliminary missions of specialized public health institutions, such as monitoring and managing chronic disease among the older adults and managing maternal health. There was a stronger correlation between population structure and efficiency in the northeast of China. The northeast has witnessed a huge labor force exodus over the past 10 years, leaving the most vulnerable populations such as the older adults, children, and women at home, which has posed huge challenges for the local public health system (82). Besides, Zhu ZK et al. disclosed the unequable accessibility to qualified public health services between migrant workers and local residents (83). Therefore, it is necessary for public health system to give priority to vulnerable population, such as the older adults, pregnant, children and migrant population.

The volume of NO_x_ emission was positively correlated with the efficiency of specialized public health services. Traditionally, air pollutants are related to dozens of diseases, such as respiratory diseases, infectious diseases, and cancers (84, 85). Therefore, larger NOx emission would bring about more public health problems, leading to greater demands and utilization of existing health resources. The coefficients turned into insignificant in all provinces since 2018, which might ascribe to the achievements in pollution abatement.

Government intervention is a powerful instrument for improving the efficiency of public health system. Since pure technical efficiency is the major trouble for the progress of specialized public health facilities, government could design and launch reforms in the management system. The integration of curative medical and preventive public health services can prompt the performance of each system (86). Therefore, central and provincial governments should take effective actions such as coordination in budget management, to alter the isolated conditions between curative and preventive systems (87). Moreover, a reasonable integration of public health services items could eliminate unnecessary duplications in the workflow and then boost the efficiency of specialized public health facilities (88). As evidenced, the adoption of hospital information system has brought about remarkable improvements in hospitals’ efficiency (89). Similarly, specialized public health facilities might also benefit from a well-equipped digital public health platform which enables effective data integration, sharing and utilization.

This study has remarkable contributions for promoting the performance of specialized public health in China. Even though, there are some limitations. Firstly, we only included data from 2017 to 2019. During the COVID-19 period, some primary responsibilities of specialized public health facilities have been undertaken by other types of health entities, such as hospitals, and primary health care institutions. Besides, the implementation of lockdowns has profound impacts on public health services’ provision, such as maternal and child health management. Moreover, the reported data of infectious disease present with larger fluctuations due to the pandemic. With these considerations, we did not include the data during COVID-19 period to avoid the bias in input and output variables. The routines functions of specialized public health have basically restored in 2023, and several reforms have been made during this period. Therefore, in the future, it would be meaningful to evaluate the efficiency of specialized public health facilities in post-pandemic era with a longer time series data. Besides, the analysis was based on the provincial data due to data accessibility. If possible, it is highly recommended to measure and compare the efficiency of Chinese public health system at municipal or even county level for more nuanced findings and implications. Finally, we have identified several significant inputs and outputs associated with public health services base on literature review. Meanwhile, it is acknowledged that more intricate and comprehensive metrics could be developed to further evaluate the efficiency of public health services.

## Conclusion

Based on the data of 31 provinces from 2017 to 2019, this paper constructed a super SBM DEA and GWR models to assess the efficiency of specialized public health services and explored its influential factors. According to our findings, specialized public health services in China have not achieved full efficiency, with larger improving space in the pure technical efficiency. The efficiency also presents with substantial differences across regions and provinces. Importantly, we demonstrate the impacts of social environmental factors on the efficiency by means of GWR, which reveals the spatial heterogeneity in the impact of each explanatory element. Apart from these meaningful findings, the time period in our study merely included 3 years from 2017 to 2019, which is a potential limitation. However, we did not include the yearly data during COVID period, given the significant fluctuations in the provision and outcomes of public health services in this period, which may lead to some bias in the estimated efficiency. In future researches, it is valuable to assess the performance of public health services using longer time series data in the post-pandemic era.

## Data Availability

The original contributions presented in the study are included in the article/Supplementary material, further inquiries can be directed to the corresponding authors.
