# Supplementary material for: How efficient are specialized public health services in China? A data envelopment analysis and geographically weighted regression approach
Source: Front Public Health. 2025 Feb 12;13:1481402. doi: 10.3389/fpubh.2025.1481402 (PMC11861560; doi:10.3389/fpubh.2025.1481402)
Supplement: Supplementary file 1 [file Image_1.pdf]

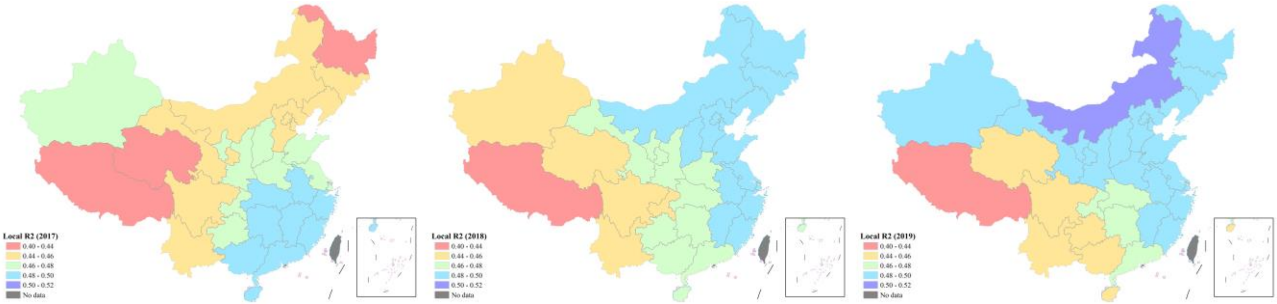

Figure S1 The spatial distribution of local R^2 of the GWR model in 2017, 2018 and 2019. These figures were drawn based on the standard map (No. GS (2019)1822) from Ministry of Natural Resources of the People's Republic of China.
