# Supplementary material for: How efficient are specialized public health services in China? A data envelopment analysis and geographically weighted regression approach
Source: Front Public Health. 2025 Feb 12;13:1481402. doi: 10.3389/fpubh.2025.1481402 (PMC11861560; doi:10.3389/fpubh.2025.1481402)
Supplement: Supplementary file 2 [file Table_1.DOCX]

**Table S1** Average technical, pure technical and scale efficiency of specialized public health services by provinces from 2017 to 2019

| **Province** | **Technical efficiency** | **Pure technical efficiency** | **Scale efficiency** |
| --- | --- | --- | --- |
| East: |  |  |  |
| Beijing | 0.6120 | 0.9043 | 0.7205 |
| Fujian | 0.5785 | 0.5902 | 0.9810 |
| Guangdong | 0.4260 | 0.4524 | 0.9388 |
| Hainan | 0.3792 | 0.3944 | 0.9603 |
| Hebei | 0.6654 | 0.6871 | 0.9680 |
| Jiangsu | 1.7429 | 1.4427 | 1.2342 |
| Liaoning | 1.1992 | 1.2188 | 0.9852 |
| Shandong | 0.5684 | 0.5763 | 0.9868 |
| Shanghai | 0.9978 | 1.7856 | 0.5728 |
| Tianjin | 1.3216 | 1.5183 | 0.8974 |
| Zhejiang | 0.6521 | 0.9561 | 0.7111 |
| Center: |  |  |  |
| Anhui | 0.9484 | 0.9851 | 0.9637 |
| Heilongjiang | 0.6907 | 0.7102 | 0.9749 |
| Henan | 0.4687 | 0.5083 | 0.9200 |
| Hubei | 0.4211 | 0.4490 | 0.9360 |
| Hunan | 0.4303 | 0.4964 | 0.8676 |
| Jiangsu | 0.4825 | 0.5080 | 0.9465 |
| Jilin | 0.8511 | 1.0543 | 0.8595 |
| Shanxi | 0.6560 | 0.6975 | 0.9409 |
| West: |  |  |  |
| Chongqing | 0.6355 | 0.6418 | 0.9910 |
| Gansu | 0.5581 | 0.5618 | 0.9932 |
| Guangxi | 0.4161 | 0.6263 | 0.7737 |
| Guizhou | 0.5322 | 0.5412 | 0.9844 |
| Inner Mongolia | 0.4710 | 0.5070 | 0.9268 |
| Ningxia | 0.4798 | 0.5236 | 0.9161 |
| Qinghai | 0.6060 | 0.6077 | 0.9971 |
| Shaanxi | 0.5295 | 0.5600 | 0.9451 |
| Sichuan | 0.5602 | 0.6009 | 0.9293 |
| Tibet | 0.4288 | 0.5470 | 0.7840 |
| Xinjiang | 0.5806 | 0.5940 | 0.9784 |
| Yunnan | 0.4727 | 0.4956 | 0.9530 |
