# Supplementary material for: How efficient are specialized public health services in China? A data envelopment analysis and geographically weighted regression approach
Source: Front Public Health. 2025 Feb 12;13:1481402. doi: 10.3389/fpubh.2025.1481402 (PMC11861560; doi:10.3389/fpubh.2025.1481402)
Supplement: Supplementary file 3 [file Table_2.DOCX]

**Table S2** Results of stepwise regression

| **Explanatory variables** | **Coefficients for standardized variables** |
| --- | --- |
| Ratio of elderly population | 0.483^***^ |
| Sex ratio | 0.273^**^ |
| Proportion of public health expenditure | -0.471^***^ |
| Total volume of Nitrogen Oxides emission | 0.185^**^ |
| Intercept | 2.724E-16 |
| Adjusted R^2^ | 0.464 |
| F-statistics | 20.882^***^ |
| Durbin-Watson | 2.387 |

Note: ^***^ indicates significance at the 1% level; ^**^indicates significance at the 5% level.
