# Supplementary material for: How efficient are specialized public health services in China? A data envelopment analysis and geographically weighted regression approach
Source: Front Public Health. 2025 Feb 12;13:1481402. doi: 10.3389/fpubh.2025.1481402 (PMC11861560; doi:10.3389/fpubh.2025.1481402)
Supplement: Supplementary file 4 [file Table_3.DOCX]

**Table S3** Global Moran’s I index of technical efficiency

| **Year** | **Moran’s I** | **Z** | **P** |
| --- | --- | --- | --- |
| 2017 | 0.2251 | 3.0703 | 0.0021 |
| 2018 | 0.2281 | 3.1068 | 0.0019 |
| 2019 | 0.1767 | 2.4855 | 0.0129 |
