# Supplementary material for: How efficient are specialized public health services in China? A data envelopment analysis and geographically weighted regression approach
Source: Front Public Health. 2025 Feb 12;13:1481402. doi: 10.3389/fpubh.2025.1481402 (PMC11861560; doi:10.3389/fpubh.2025.1481402)
Supplement: Supplementary file 5 [file Table_4.DOCX]

**Table S4** Results of GWR regression, 2017-2019

| **Parameter** | **2017** | | |  | **2018** | | |  | **2019** | | |
| --- | --- | --- | --- | --- | --- | --- | --- | --- | --- | --- | --- |
|  | Min | Median | Max |  | Min | Median | Max |  | Min | Median | Max |
| Ratio of elderly population | 0.3655 | 0.4613 | 0.5651 |  | 0.3311 | 0.4793 | 0.6480 |  | 0.3218 | 0.4257 | 0.6604 |
| Sex ratio | 0.1486 | 0.2540 | 0.3867 |  | 0.2162 | 0.3441 | 0.4703 |  | 0.1968 | 0.2864 | 0.3888 |
| Proportion of public health expenditure | -0.6031 | -0.5480 | -0.3002 |  | -0.5416 | -0.5096 | -0.4079 |  | -0.4929 | -0.4524 | -0.2782 |
| Total volume of Nitrogen Oxides emission | 0.0545 | 0.2773 | 0.2921 |  | 0.0638 | 0.2089 | 0.2492 |  | -0.0209 | 0.1691 | 0.2357 |
| AICc | 88.0655 | | |  | 87.8980 | | |  | 87.0437 | | |
| R^2^ | 0.5266 | | |  | 0.5406 | | |  | 0.5492 | | |
| Adjusted R^2^ | 0.3574 | | |  | 0.3685 | | |  | 0.3850 | | |
