# Supplementary material for: How efficient are specialized public health services in China? A data envelopment analysis and geographically weighted regression approach
Source: Front Public Health. 2025 Feb 12;13:1481402. doi: 10.3389/fpubh.2025.1481402 (PMC11861560; doi:10.3389/fpubh.2025.1481402)
Supplement: Supplementary file 6 [file Table_5.DOCX]

**Table S5** Global Moran’s I index of the standard residual from the GWR model

| Year | Moran’s I | Z | P |
| --- | --- | --- | --- |
| 2017 | -0.0918 | -0.6703 | 0.5027 |
| 2018 | -0.0784 | -0.5207 | 0.6026 |
| 2019 | -0.1315 | -1.1347 | 0.2565 |
