# Supplementary material for: How efficient are specialized public health services in China? A data envelopment analysis and geographically weighted regression approach
Source: Front Public Health. 2025 Feb 12;13:1481402. doi: 10.3389/fpubh.2025.1481402 (PMC11861560; doi:10.3389/fpubh.2025.1481402)
Supplement: Supplementary file 7 [file Table_6.DOCX]

**Table S6** Return to scale of specialized public health facilities in 31 provinces

| **Categories** | **2017** | **2018** | **2019** |
| --- | --- | --- | --- |
| Decreasing returns to scale | 3 | 16 | 17 |
| Increasing returns to scale | 28 | 15 | 14 |
| Total amount | 31 | 31 | 31 |
